# Visceral fat index: a novel predictor for coronary collateral circulation

**DOI:** 10.20945/2359-3997000000218

**Published:** 2020-03-18

**Authors:** Yasin Sahinturk, Selcuk Kucukseymen, Rauf Avci, Ayse Akarsu, Basak Oguz Yolcular, Gokhan Koker, Abdullah Tokuc, Nermin Bayar, Sakir Arslan

**Affiliations:** 1 Departments of Internal Medicine University of Health Sciences Antalya Training and Research Hospital Antalya Turkey Departments of Internal Medicine, University of Health Sciences Antalya Training and Research Hospital, Antalya, Turkey; 2 Departments of Cardiology University of Health Sciences Antalya Training and Research Hospital Antalya Turkey Departments of Cardiology, University of Health Sciences Antalya Training and Research Hospital, Antalya, Turkey; 3 Department of Biostatistics Akdeniz University Antalya Turkey Department of Biostatistics Akdeniz University, Antalya, Turkey

**Keywords:** Obstructive coronary artery disease, coronary collateral circulation, visceral adiposity, hypertension, body mass index

## Abstract

**Objective:**

This study was designed to investigate the role of visceral adiposity along with other clinical parameters in predicting poor coronary collateral circulation (CCC) among patients with severe obstructive coronary artery disease (CAD).

**Subjects and methods:**

A total of 135 patients with severe obstructive CAD and good (n = 70) or poor (n = 65) CCC were included. Data on angiographically detected CCC, the quality criteria for CCC (Rentrop scores) and visceral fat index (VFI) obtained via bioelectrical impedance were compared between good and poor CCC groups. Independent predictors of poor CCC, the correlation between VFI and Rentrop score and the role of VFI in the identification of CCC were analyzed.

**Results:**

A significant negative correlation was noted between VFI and Rentrop scores (r = -0.668, < 0.001). The presence of hypertension (OR 4.244, 95% CI 1.184 to 15.211, p = 0.026) and higher VFI (OR 1.955, 95% CI 1.342 to 2.848, p < 0.001) were shown to be independent predictors of an increased risk for poor CCC. ROC analysis revealed a VFI > 9 (AUC [area under the curve] (95% CI): 0.898 (0.834-0.943), p < 0.0001) to be a potential predictor of poor CCC with a sensitivity of 95.38% and specificity of 85.71%.

**Conclusion:**

In conclusion, our findings revealed comorbid hypertension and higher VFI to significantly predict the risk of poor CCC in patients with severe obstructive CAD.

## INTRODUCTION

Coronary collateral circulation (CCC) is a physiological adaptation that serves as a natural bypass system to restore blood flow in myocardium jeopardized by stenosis or the occlusion of a coronary vessel (1,2). Well-developed CCC is considered to protect myocardial function by limiting the infarct size and improving survival after transient or permanent coronary obstruction in patients with coronary artery disease (CAD) (2-7). Thus, the identification of factors associated with CCC development is considered to be of great clinical significance (8,9).

Given that coronary collateral growth depends on intact vascular endothelium and endothelial function, factors contributing to vascular dysfunction have also been suggested to be associated with poor CCC (10-12). Accordingly, genetic factors, age, degree of coronary artery stenosis, presence of total occlusion, myocardial ischemia, severe multivessel coronary stenosis, physical exercise, smoking, obesity, hyperlipidemia, diabetes mellitus and metabolic syndrome have been proposed to be related to coronary collateral development in several studies (10,13-20).

However, while many pathogenic insults have been proposed, given the existence of conflicting data, the exact mechanism of CCC has not yet been completely described (7,11,13,20-22).

Obesity is considered an independent risk factor for cardiovascular disease (CVD), while endothelial dysfunction and subclinical inflammation, along with cardiovascular risk status, have been associated with an increased incidence of CV events in obese patients (23-25). The increasing demand due to elevated cardiac output combined with the risk of down perfusion via large artery stenosis-dependent flow restriction is considered the main clinical dilemma related to cardiac health in obese patients (26).

Visceral adiposity, rather than general obesity, has been demonstrated in several studies to be more strongly related to complications such as risk of CVD, insulin resistance and type 2 diabetes, while visceral fat was also shown to have greater pro-inflammatory characteristics than subcutaneous fat (27-29). However, no studies to date have investigated the potential role of visceral adiposity in coronary collateral development in patients with severe obstructive CAD. The present study was therefore designed to investigate the role of visceral adiposity along with other clinical parameters in predicting poor CCC among patients with severe obstructive CAD.

## SUBJECTS AND METHODS

### Study population

A total of 135 patients (mean (SD) age: 59.3 (10.3) years, 82.2% were male) with CAD who had total occlusion of at least one major coronary artery demonstrated by invasive coronary angiography were included in this prospective study conducted between 2017 and 2018. Patients were divided into two groups based on collateral degree: the good CCC group (Rentrop grades 2-3, n = 70) and the poor CCC group (Rentrop grades 0-1, n = 65) according to the Rentrop Cohen classification (30). Patients who were suffering from angina-type chest pain for 3-6 months and who had angiography due to stable angina pectoris that resulted in findings consistent with chronic total occlusion in a coronary artery were included in the study. Patients with a past history of CAD, coronary revascularization, chronic alcohol usage, hepatitis B and C infections or chronic kidney disease and patients with diabetes on pioglitazone treatment were excluded from the study.

The study was conducted in full accordance with the local Good Clinical Practice guidelines and current legislations, and permission was obtained from the Clinical Research and Ethics Committee of University of Health Sciences Antalya Training and Research Hospital for the use of patient data for publication purposes (date of approval 19/1/2017; reference number/protocol no: 2/2).

### Study parameters

Data on patient demographics (age, sex), smoking and alcohol consumption, comorbidities (diabetes, hypertension), concomitant medications, CAD characteristics (type and number of occluded coronary arteries), anthropometrics [body mass index (BMI, kg/m^2^), muscle mass, fat mass, visceral fat index (VFI)], blood biochemistry [total cholesterol, triglycerides, high-density lipoprotein (HDL), low-density lipoprotein (LDL), creatinine], hemogram [neutrophil, lymphocyte, thrombocyte, white blood cell (WBC) counts, hemoglobin] and inflammatory markers [C-reactive protein (CRP), neutrophil-to-lymphocyte ratio (NLR), platelet-to-lymphocyte ratio (PLR), neutrophil-to-platelet ratio (NPR)] were recorded for each patient and compared between the good and poor CCC groups.

### Coronary angiography and collateral grading

Coronary angiography was performed with the Judkins technique and was evaluated in terms of total coronary occlusion by two experienced cardiologists to determine CCC. Collateral flow was graded using the Rentrop scoring system (0: no visible filling of any collateral channels, 1: filling of the small side branches, 2: partial collateral filling of the epicardial artery, 3: complete collateral filling of the epicardial artery) (30). Patients were then classified as having poor CCC (Rentrop grades 0-1) or good CCC (Rentrop grades 2-3).

### Blood tests for inflammatory markers

Serum CRP levels were measured as high-sensitivity CRP by an immunoturbidimetric assay using the C-Reactive Protein High Sensitivity reagent (Beckman Coulter, Inc., Fullerton, CA, USA; limit of detection, 0.08 mg/L). The NLR, PLR and NPR were calculated as the ratio of the absolute neutrophil or platelet count and the absolute lymphocyte count, provided by the differential white blood cell count (WBC) measured by a Sysmex XE-2100 automated hematology analyzer (Sysmex Corporation).

### Anthropometrics

Body weight (kg) and height (m) were measured with the participants in a standing position, and BMI (kg/m^2^) was calculated at the time of body composition measurement. Elementary body composition was measured using a direct segmental multifrequency bioelectrical impedance analyzer (InBody770) to identify total fat mass and total muscle mass. Bioimpedance measurements were performed with attention to the state of hydration (lack of ascites, pleural effusion and pretibial edema), time (on the morning of the first hospitalization day) and clothes worn (with standard clothing and no personal belongings). VFI was assessed to measure visceral fat composition via bioelectrical impendence methods using Omron body fat and weight measurement devices (V-body HBF-371; Omron, Kyoto, Japan). To obtain the data of VFI from the resistance between the two hands and feet, an individual’s height, weight, age and sex were input into the instrument and the subjects stood on the footplate barefoot and holding the handle electrodes in both hands horizontally forward. The presence of visceral fat was categorized by the threshold values (≤ 9: normal, 10-14: high, 15-30: very high) provided by the manufacturer with the scale, as graded arbitrarily from 0 to 30.

### Statistical analysis

Sample size calculation was performed using the G*Power 3.1.9 program, which revealed that at least 62 patients (31 patients in each group) needed to be included in the study, based on a power of 80% and a type I error of 0.05, assuming a correlation coefficient of ≥ 0.3 between variables. A post hoc power analysis using G Power 3.1 revealed 99.9% statistical power with a 0.05 alpha level and d = 1.77.

Statistical analysis was performed using IBM SPSS Statistics for Windows, Version 22.0 (IBM Corp., Armonk, NY). Fisher’s exact test and Pearson chi-square analysis were performed for categorical variables. The normality assumptions of the analysis of the two-group measurement differences were controlled by the Shapiro–Wilk test. The Mann–Whitney U test was used for the analysis of nonnormally distributed numerical data, while Student’s t test was used for normally distributed data. A Spearman correlation test was performed to test relationships of ordinal or quantitative variables with nonnormal distributions. The ROC curve was plotted to determine the performance of VFI in the identification of CCC by calculating the area under the curve (AUC) values and the ideal cut-off value via ROC analysis. Univariate logistic regression analysis was performed to identify potential risk factors for poor CCC. Variables with a p value < 0.05 in the univariate analysis were analyzed further in a multivariate regression model to identify statistically significant risk factors for poor CCC.

Data are expressed as the mean (standard deviation, SD) or median (min-max), as appropriate. p < 0.05 was considered statistically significant.

## RESULTS

### Demographics and clinical characteristics of the patients with good vs. poor CCC

Overall, CCC was good in 70 (51.9%) patients and poor in 65 (48.1%) patients with occlusion of 1-2 coronary arteries, including the right coronary artery in most of the patients.

Poor vs. good CCC was associated with older patient age (63.3 (9.3) vs. 55.7 (9.9) years, p < 0.001), higher male percentage (90.8 vs. 74.3%, p = 0.012), higher rates of hypertension (76.9 vs. 52.9%, p = 0.004), ACE/ARB (84.6 vs. 58.6%, p = 0.001), beta blockers (80.0 vs. 62.9%, p = 0.028) and anticoagulant use (16.9 vs. 1.4%, p = 0.002) (Table 1).

**Table 1:** Demographics and clinical characteristics

		Total (n = 135)	Coronary collateral circulation		p value

			Poor (n = 65)	Good (n = 70)	
**Demographics**					
Age (years), mean (SD)		59.3 (10.3)	63.3 (9.3)	55.7 (9.9)	**< 0.001**^**1**^
Sex (Male/Female), n (%)		111 (82.2)/24 (17.8)	59 (90.8)/6 (9.2)	52 (74.3)/18 (25.7)	**0.012^3^**
**Comorbidities, n (%)**					
Smoking		36 (26.7)	15 (23.1)	21 (30)	0.363^3^
Alcohol		15 (11.1)	5 (7.7)	10 (14.3)	0.223^3^
Diabetes		57 (42.2)	33 (50.8)	24 (34.3)	0.053^3^
Hypertension		87 (64.4)	50 (76.9)	37 (52.9)	**0.004^3^**
**Concomitant medications**					
CCB, n (%)		21 (15.6)	9 (13.8)	12 (17.1)	0.597^3^
ACEi/ARB, n (%)		96 (71.1)	55 (84.6)	41 (58.6)	**0.001^3^**
Beta blocker, n (%)		96 (71.1)	52 (80.0)	44 (62.9)	**0.028^3^**
Nitrate, n (%)		9 (6.7)	5 (7.7)	4 (5.7)	0.738^3^
Statin, n (%)		129 (95.6)	63 (96.9)	66 (94.3)	0.682^3^
Antiplatelet, n (%)		129 (95.6)	60 (92.3)	69 (98.6)	0.105^3^
Anticoagulant, n (%)		12 (8.9)	11 (16.9)	1 (1.4)	**0.002^3^**
**CAD characteristics**					
Occluded coronary artery, n (%)					
Right coronary artery		96 (71.1)	53 (81.5)	43 (61.4)	N/A
Obtuse marginal branches 2		6 (4.4)	4 (6.2)	2 (2.9)	
Circumflex		12 (8.9)	2 (3.1)	10 (14.3)	
Left anterior descending artery		18 (13.3)	6 (9.2)	12 (17.1)	
Diagonal 1		3 (2.2)	0 (0)	3 (4.3)	
**Number of occluded vessels, n (%)**					
1		50 (37.0)	27 (41.5)	23 (32.9)	0.124^3^
2		52 (38.5)	26 (40.0)	26 (37.1)	
3		24 (17.8)	11 (16.9)	13 (18.6)	
4		9 (6.7)	1 (1.5)	8 (11.4)	
**Rentrop score, n (%)**					
0		3 (2.2)	3 (4.6)	0 (0)	-
1		62 (45.9)	62 (95.4)	0 (0)	
2		47 (34.8)	0 (0)	47 (67.1)	
3		23 (17)	0 (0)	23 (32.9)	
**Anthropometrics**					
BMI (kg/m^2^), mean (SD)		27.6 (3.6)	28.4 (3.7)	27.0 (3.3)	**0.026^1^**
Muscle mass, median (min-max)		55.9 (38-67.3)	56.4 (38.7-67.3)	54.4 (38-66.6)	**0.030^2^**
Fat mass, median (min-max)		20.1 (8.2-41.4)	23.0 (8.2-41.4)	19.0 (8.2-41.4)	**0.002^2^**
VFI	Median (min-max)	12 (4-23)	13.0 (7-23)	7.0 (4-23)	**<0.001^2^**
	1-9, n (%)	63 (46.7)	3 (4.6)	60 (85.7)	**<0.001^3^**
	> 9, n (%)	72 (53.3)	62 (95.4)	10 (14.3)	
**Blood biochemistry**					
Total cholesterol, median (min-max)		193 (87-336)	203 (87-336)	191 (143-336)	0.798^2^
Triglycerides, median (min-max)		173 (51-597)	175 (51-597)	151 (65-597)	0.292^2^
HDL, mean (SD)		43.2 (8.1)	44.1 (8.1)	42.3 (8.1)	0.198^1^
LDL, median (min-max)		113 (37-253)	106 (37-253)	121.5 (78-253)	0.154^2^
Creatinine, mean (SD)		1.1 (0.2)	1.1 (0.1)	1 (0.2)	**< 0.001^1^**
TG/HDL-c, median (min-max)		3.81 (1.38-13)	3.79 (1.62-13)	4.14 (1.38-11.48)	0.176^2^
HbA1c, median (min-max)		5.7 (4-11.5)	6 (4.2-11.5)	5.5 (4-11.1)	0.203^2^
**Hemogram**					
Neutrophil, median (min-max)		5000 (2100-8700)	4600 (2600-7700)	5150 (2100-8700)	**0.005^2^**
Lymphocyte, median (min-max)		2500 (700-4600)	2200 (700-3800)	2600 (1300-4600)	**0.001^2^**
Thrombocyte, median (min-max)		265000 (100000-370000)	247000 (100000-370000)	275500 (143000-370000)	**0.010^2^**
WBC, median (min-max)		8500 (4100-12700)	7800 (4100-11700)	9300 (5700-12700)	**< 0.001^2^**
Hemoglobin, median (min-max)		14.2 (8.5-17.6)	14.2 (8.5-17.6)	14.5 (9.9-16.2)	0.425^2^
**Inflammatory markers, median (min-max)**					
CRP		15 (2-101)	12 (2-101)	15 (2-101)	0.396^2^
NLR		2.1 (0.81-5.86)	2.19 (1.11-5.86)	1.92 (0.81-3.92)	0.155^2^
PLR		0.01 (0.003-0.02)	0.01 (0.003-0.02)	0.01 (0.005-0.02)	0.198^2^
NPR		0.02 (0.009-0.031)	0.02 (0.01-0.031)	0.02 (0.009-0.031)	0.115^2^

ACEi/ARB: angiotensin-converting enzyme inhibitor/angiotensin II receptor blockers; BMI: body mass index; CCB: calcium channel blocker; CRP: C-reactive protein; HDL: high-density lipoprotein; LDL: low-density lipoprotein; N/A: not applicable; NLR: neutrophil-to-lymphocyte ratio; NPR: neutrophil-to-platelet ratio; PLR: platelet-to-lymphocyte ratio; VFI: visceral fat index; WBC: white blood cell.

^1^ Student’s t test; ^2^ Mann-Whitney U test; ^3^ Chi square test.

Median (min-max) levels of BMI (28.4 (3.7) vs. 27.0 (3.3) kg/m^2^, p = 0.026), muscle mass (56.4 (38.7-67.3) vs. 54.4 (38-66.6), p = 0.030), fat mass (23.0 (8.2-41.4) vs. 19.0 (8.2-41.4), p = 0.002) and VFI (13.0 (7-23) vs. 7.0 (4-23), p < 0.001) were significantly higher in patients with poor vs. good CCC. Compared to those in the good CCC group, a significantly higher percentage of patients in the poor CCC group had VFI > 9 (95.4 vs. 14.3%, p < 0.001) (Table 1).

The median (min-max) neutrophil (4600 (2600-7700) vs. 5150(2100-8700) /mm^3^, p = 0.005), lymphocyte (2200 (700-3800) vs. 2600 (1300-4600)/mm^3^, p = 0.001), thrombocyte (247000 (100000-370000) vs. 275500 (143000-370000)/mm^3^, p = 0.010) and WBC (7800 (4100-11700) vs. 9300 (5700-12700)/mm^3^, p < 0.001) counts were significantly lower in the poor CCC group than in the good CCC group.

The poor vs. good CCC groups were similar in terms of the lipid profile, TG-HDL-c ratio and inflammatory markers, including CRP, NLR, PLR and NPR (Table 1).

### ROC analysis

**Figure 1:**
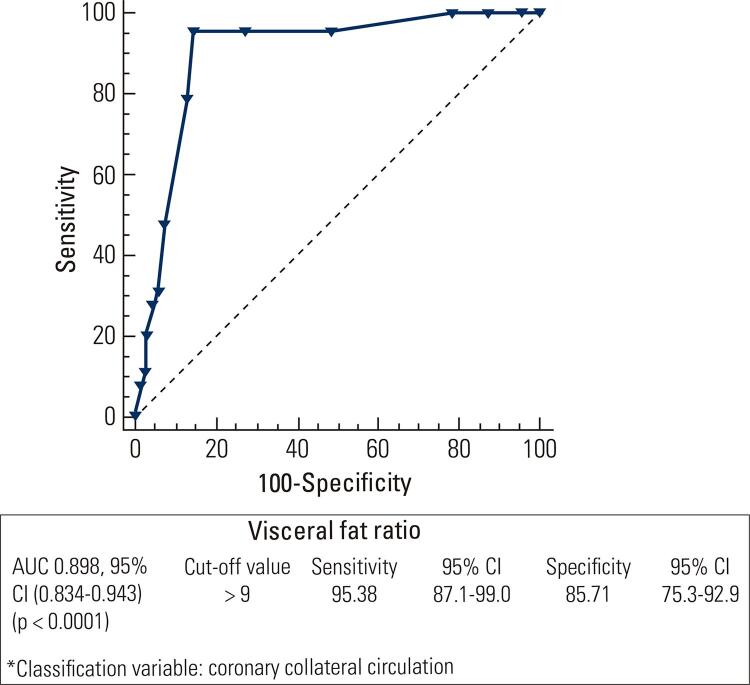
ROC curve analysis of the role of VFI in the prediction of coronary collateral circulation. AUC: area under the curve.

There was a significant negative correlation between VFI and Rentrop scores (r = -0.668, < 0.001). ROC analysis revealed that VFI > 9 (AUC (95% CI): 0.898 (0.834-0.943), p < 0.0001) was a potential marker of poor CCC with a sensitivity of 95.38% and specificity of 85.71% (Figure 1).

### Logistic regression analysis for independent predictors of poor CCC

Among the variables significantly associated with poor CCC in the univariate analysis [older age, male sex, hypertension, BMI > 25 kg/m^2^, higher muscle mass, fat mass and VFI], only the presence of hypertension (OR 4.244, 95% CI 1.184 to 15.211, p = 0.026) and higher VFI (OR 1.955, 95% CI 1.342 to 2.848, p < 0.001) were shown to be independent predictors of an increased risk for poor CCC (Table 2).

**Table 2:** Univariate and multivariate logistic regression analyses for independent predictors of poor CCC

Variables	Univariate analysis		Multivariate analysis	

	OR (95% CI)	p value	OR (95% CI)	p value
Age	1.084 (1.042-1.128)	**< 0.001**	0.988 (0.896-1.09)	0.816
Sex (M/F)	3.404 (1.257-9.219)	**0.016**	0.051 (0.001-2.996)	0.152
BMI>25	2.691 (1.164-6.224)	**0.021**	0.988 (0.146-6.696)	0.990
DM	1.977 (0.989-3.952)	0.054	2.262 (0.666-7.68)	0.191
Hypertension	2.973 (1.413-6.255)	**0.004**	4.088 (1.154-14.482)	**0.029**
Muscle mass	1.057 (1.011-1.105)	**0.014**	1.084 (0.928-1.265)	0.310
Fat mass	1.084 (1.028-1.142)	**0.003**	0.82 (0.667-1.009)	0.061
VFI	1.691 (1.437-1.990)	**< 0.001**	2.054 (1.403-3.005)	**< 0.001**

BMI: body mass index; CI: confidence interval; DM: diabetes mellitus; OR: odds ratio; VFI: visceral fat index.

## DISCUSSION

Our findings in a cohort of CAD patients with total coronary occlusion revealed the presence of hypertension and higher VFI to be significant determinants of poor CCC development. To the best of our knowledge, the present study is the first clinical study to identify an increase in visceral fat as a potential independent predictor of poor collateral development.

The presence of metabolic syndrome (31-33), an increasing number of components of metabolic syndrome (33,34), aging (15), obesity (18) and the presence of hypertension (20,35) have been associated with an increased risk of impaired collateral development among patients with obstructive CAD. However, there is controversy regarding the impact of diabetes or smoking on collateral development, with associations of diabetes (10,11) and nonsmoking (20) with a higher risk of poor collateral development shown in some studies, whereas no associations of diabetes (36) or smoking (13) with collateral growth and an association of inflammation with either promoted or inhibited collateral growth (37) have been reported in other studies.

In addition, a positive association of leukocytes (38) and inflammatory cytokines (39) with collateral development has also been documented in healthy animal models. Higher neutrophil and monocyte count (22) and mean platelet volume (40) and lower lymphocyte count (22) upon admission were reported to be associated with better collateral networks in patients with acute coronary syndrome, while elevated hs-CRP levels (11,41,42) have been associated with an increased risk of impaired collateral development among patients with obstructive CAD.

Our findings revealed no association of inflammatory markers (CRP, NLR, PLR), lipid profile (LDL, HDL, triglycerides, total cholesterol, TG-HDL-c ratio), diabetes or smoking with coronary collateral development. Although older age, male sex, BMI >25 kg/m^2^ and higher muscle and fat mass were also among the factors significantly associated with poor CCC in the univariate analysis, multivariate analysis revealed hypertension and higher VFI to be the only independent predictors of poor CCC.

Visceral adipose tissue is a metabolically active organ, and excess levels of visceral adipose tissue, regardless of BMI, is considered an independent risk factor for diabetogenic and atherogenic abnormalities (43-45). Visceral fat was also shown to be a stronger risk factor for CVD than BMI or other fat depots (27). Thus, the assessment of both general and abdominal adiposity has been suggested to provide a better assessment of mortality risk, particularly among people with a lower BMI (46).

VFI is considered to be an accurate and reliable method for evaluating visceral adiposity and has been shown to be highly relevant to visceral fat measured by the gold standard methods (47-50). In our study, measurements were based on bioimpedance analysis, which is a low cost, easily applied technique associated with a globally accepted standardization and a low risk of bias if measurements are performed appropriately. To the best of our knowledge, the present study is the first clinical study to show an increase in visceral fat as a potential independent predictor of poor collateral development. In addition to its significant negative correlation with Rentrop scores, VFI (cut off value > 9) was also shown to predict the likelihood of poor CCC with a sensitivity of 95.38% and a specificity of 85.71%. Likewise, in a past study comparing the levels of pericardial fat index, paracardial fat index, and visceral fat index in patients with vs. without CAD, the levels of these fat indices were shown to be significantly higher in the CAD group and positively correlated with the number of significantly stenosed coronary vessels and the Gensini score in CAD patients (51).

Thus, in light of clinical entities, such as “metabolically healthy” obesity phenotypes and the “obesity paradox” (52), our findings emphasize the consideration of visceral adiposity rather than BMI as a more adequate indicator of collateral development, which plays a major role in cardiovascular outcomes and survival (3,5,7,13). Similarly, in a community-based cross-sectional study regarding obesity indices and myocardial infarction, VFI (optimal cut-off values of 15 for males and 10 for females) was shown to be associated with higher predictive power and higher sensitivity than BMI and waist circumference in the identification of CVD risk in both sexes (53).

In addition, the significant role of VFI in the prediction of poor CCC seems to indicate the likelihood of considering visceral adiposity as a potential target, with maintenance of VFI values below 9 suggested for the prevention of poor collateral growth in patients with CV risk factors in the long term.

Hypertension was shown to be a strong independent risk factor for poor CCC in our cohort of patients with total coronary occlusion. This finding seems to be in line with the association of hypertension with an increased risk of impaired collateral development in patients with coronary occlusion reported in past studies (20,35). Notably, in a past study among patients with single chronic total occlusion of the coronary artery, a J-shaped relationship was noted between diastolic blood pressure (DBP) and CCC (54). The authors indicated a decrease in the incidence of poor CCC as DBP increased, while poor CCC increased as DBP increased further to >95 mmHg (54). Given that most coronary blood flow to the myocardium occurs in diastole, the authors suggested that DBP may influence the tangential fluid shear stress of the endothelial surface by modulating the coronary artery blood flow velocity in diastole, thereby affecting the development of CCC (54). The authors also emphasized that excessively high DBP might lead to stenosis in the CCC-fed coronary artery, which in turn would decrease the perfusion of CCC (54).

### Study limitations

The main limitation of this study seems to be the assessment of visceral fat based on VFI, without the inclusion of gold-standard adiposity measurements such as micromagnetic resonance imaging (µMRI) and microcomputed tomography (µCT), which otherwise would extend the knowledge achieved in the current study. However, µCT is an expensive method with considerable radiation risk that limits its use in every patient and only for the purpose of visceral fat measurement, while µMRI is a very expensive and time-consuming method that is not appropriate for use in visceral fat measurement. Accordingly, given the limitations of the other two techniques, the use of bioimpedance analysis seems to be a clinically feasible approach for visceral fat measurement based on the extensive literature support. Nonetheless, given the restricted amount of data available on the potential role of visceral adiposity in collateral development in patients with coronary occlusion, our findings represent a valuable contribution to the literature.

In conclusion, our findings revealed that comorbid hypertension and higher VFI (cut off value > 9) significantly predict the risk of poor CCC with a sensitivity of 95.38% and specificity of 85.71%. Thus, there may be a potential clinical role of visceral adiposity assessment in the diagnosis of poor collateral development, and we may consider visceral adiposity as a potential target for preventing poor collateral circulation in patients with established CV risk in the long term.
